# Exploring transitions in care from pulmonary rehabilitation to home for persons with chronic obstructive pulmonary disease: A descriptive qualitative study

**DOI:** 10.1111/hex.13012

**Published:** 2020-01-01

**Authors:** Jonathan Miranda, Danielle Underwood, Miranda Kuepfer‐Thomas, Drew Coulson, Andy Chansoo Park, Stacey J. Butler, Roger Goldstein, Dina Brooks, Amanda C. Everall, Sara J. T. Guilcher

**Affiliations:** ^1^ Department of Physical Therapy University of Toronto Toronto ON Canada; ^2^ Department of Respiratory Medicine West Park Healthcare Centre York ON Canada; ^3^ Rehabilitation Sciences Institute University of Toronto Toronto ON Canada; ^4^ School of Rehab Science McMaster University Hamilton ON Canada; ^5^ Leslie Dan Faculty of Pharmacy University of Toronto Toronto ON Canada; ^6^ Institute of Health Policy, Management and Evaluation University of Toronto Toronto ON Canada

**Keywords:** chronic obstructive, health personnel, pulmonary disease, rehabilitation, self‐management, social support, transitional care

## Abstract

**Background:**

Individuals with chronic obstructive pulmonary disease (COPD) often experience high health‐care utilization following pulmonary rehabilitation, suggesting suboptimal transitions to home.

**Objective:**

To understand the experiences of persons with COPD and health‐care professionals regarding transitions from pulmonary rehabilitation to home, including factors impacting these transitions.

**Design:**

A descriptive qualitative study.

**Setting and participants:**

Health‐care professionals working at, and persons with COPD who attended, an inpatient or outpatient pulmonary rehabilitation programme at one large, urban health‐care centre. The centre is located in Ontario, Canada.

**Main variable studied:**

Experiences of participants with care transitions between pulmonary rehabilitation and home. Semi‐structured interviews were audio‐recorded, transcribed verbatim, and thematically analysed.

**Results:**

Ten patients and eight health‐care professionals participated. Four main themes were identified around the overall experiences with pulmonary rehabilitation and transitions to home: (a) pulmonary rehabilitation as a safe environment; (b) pulmonary rehabilitation as a highly structured environment; (c) contrasting perceptions of the role of pulmonary rehabilitation; and (d) dependency on pulmonary rehabilitation programmes. Persons with COPD and health‐care professionals identified three key factors that influenced this transition: (a) patients' social support, (b) application of self‐management strategies prior to discharge, and (c) patients' physical and mental health.

**Conclusion:**

Participants agreed that some patients with COPD experienced suboptimal transitions from pulmonary rehabilitation to home that were characterized by suboptimal self‐management. Further research is needed to develop and evaluate interventions to improve transitions. Such interventions should include strategies to elicit long‐term behaviour change to assist patients when they return into the community.

## 1 INTRODUCTION

Chronic obstructive pulmonary disease (COPD) is a progressive, life‐threatening disease that is associated with many comorbidities, high health‐care utilization, and high mortality rates.^1^, ^2^, ^3^, ^4^ Moderate‐to‐severe COPD has been estimated to affect nearly 65 million individuals worldwide.^5^ Because of their high health‐care utilization, persons with COPD often experience transitions in care as they move from one setting to another.^1^ While the goal of transitions in care is to maintain continuity of disease management regardless of the patient's location,^6^, ^7^ persons with COPD often experience suboptimal transitions.^8^, ^9^ This can lead to emergency department visits and hospital readmissions.^6^, ^7^ Within the first year following hospitalization, 18% of patients with COPD in Canada are readmitted once, while 14% are readmitted at least twice.^10^

For individuals with COPD who experience reduced activity tolerance due to dyspnoea, pulmonary rehabilitation is the recommended therapy.^11^, ^12^ Pulmonary rehabilitation is a comprehensive collection of patient‐tailored therapies that intend to provide patients with COPD the education and skills to self‐manage their disease at home.^12^ However, due to the structured design of these programmes, being discharged from pulmonary rehabilitation signifies another point of transition for persons with COPD. In an attempt to improve the transition from pulmonary rehabilitation to home, on‐going communication and continuity with health‐care professionals are commonly identified needs.^6^, ^7^, ^13^, ^14^

Another key factor in facilitating optimal transitions in care is self‐management.^15^, ^16^ Self‐management education teaches skills required for disease‐specific treatments, assists with behaviour change and promotes emotional support.^15^ Self‐management programmes have been demonstrated to improve outcomes, including decreased hospitalization admissions, among persons with COPD.^17^, ^18^ Unfortunately, the current literature suggests that patients with COPD often have difficulties applying self‐management strategies,^19^, ^20^ even after following pulmonary rehabilitation,^13^, ^21^ with minimal research on why. A 2017 qualitative study reported that health‐care providers in England identified several factors that affected self‐management for persons with COPD.^19^ These factors were complex and interrelated, including provider (knowledge, communication skills, interprofessional teams, ability to normalize self‐management into routine practice), patient (knowledge, motivation, emotional/psychological state, self‐management skills), and system‐level (service fragmentation, inconsistent pathways) factors.^19^

Prior research has focused largely on compliance to home exercise programmes post‐discharge from rehabilitation,^21^, ^22^, ^23^ or to transitions from acute care to the community.^8^, ^9^ To our knowledge, no studies have sought perspectives from health‐care professionals and persons with COPD during transition from rehabilitation to home. To address this gap, the purpose of this study was to explore the experiences transitioning from pulmonary rehabilitation to home from the perspectives of persons with COPD and health‐care professionals with a focus on identifying factors which affect this transition.

## 2 METHODS

### 2.1 Study design

A qualitative descriptive methodology was used to explore the experiences of transitions in care from pulmonary rehabilitation to home from the perspectives of persons with COPD and health‐care professionals.

### 2.2 Setting and participants

The pulmonary rehabilitation programme in this study is provided through a health‐care centre in Ontario, Canada. The programme includes a combination of exercise, self‐management education, and psycho‐social support provided by an interprofessional team (eg, respirologists, nurses, and physiotherapists). The programme can be provided both as inpatient and outpatient rehabilitation. Inpatients remain at the health‐care centre, receiving 22.5 hours of therapy and education per week. Outpatients attend twice weekly, receiving 6 hours of therapy and education per week. Following pulmonary rehabilitation, patients have the option to participate in a maintenance programme at the health‐care centre that involves 1 hour of supervised exercise once a week. Patients can remain in the maintenance programme for 6 months or longer depending on availability of space.

The participants in this study were individuals with COPD who completed pulmonary rehabilitation and health‐care professionals working at the rehabilitation health‐care centre. Participants with COPD were eligible if they (a) were diagnosed with moderate or severe COPD according to the Global Initiative for Chronic Obstructive Lung Disease guidelines,^1^ (b) had been discharged from inpatient or outpatient pulmonary rehabilitation within the past 1‐6 months, and (c) understood English. Health‐care professionals were required to (a) have a minimum of 1 year of experience working in pulmonary rehabilitation at the health‐care centre and (b) understand English. No exclusion criteria were applied.

A purposive sampling technique^24^ was used from a convenience sample of patients and health‐care professionals from the study health‐care centre. We sought both patients who attended the inpatient and outpatient programmes to compare and contrast experiences with transitions. We also sought diversity in health‐care professionals from different professions as their roles may influence their perceptions of transitioning patients to home. A staff member on site approached patients and health‐care professionals for consent to contact, and once received, the researchers contacted the potential participants for consent to participate.

### 2.3 Data collection

Two researchers completed semi‐structured interviews with patients and health‐care professionals. Interviews were audio‐recorded and conducted either in‐person or by telephone. Two interview guides (one for persons with COPD and one for health‐care professionals) with open‐ended questions and probes were used (see Tables 1 and 2). Questions were developed with considerations of the key domains from the Ideal Transitions of Care Framework^16^ to capture important aspects of an ideal transition (eg, communication, advice, and discharge planning). During the interview, participants were also asked to complete a short socio‐demographic (eg, age and sex) and/or clinical questionnaire (eg, professional role and years of experience).

**Table 1:** Interview guide for patients

Question and probes
1. Can you tell us about yourself and your background?
What are some hobbies or activities you enjoy?
Do you have family/friends who live near you?
2. What was it like going home from pulmonary rehabilitation at (Health‐care Centre)?
What emotions/feelings were you experiencing?
Did you have any concerns about being discharged?
Did anything surprise you about going home?
What were some of the goals or expectations?
3. How have your daily activities changed since pulmonary rehabilitation?
Example of good/challenging aspects?
Were you prepared for these challenges?
How has managing symptoms been?
4. What information or supports helped with going home?
Which health‐care professions were involved?
What information was useful? What wasn't?
How was the information presented?
5. What advice would you give the health‐care professionals?
Any particular profession?
6. What advice do you have for other patients?

**Table 2:** Interview guide for health‐care professionals

Question and probes
1. What is your experience in the pulmonary rehabilitation programme?
2. What are the main concerns brought up by patients as they near the end of the programme?
Are there concerns that are easy to address?
Are there concerns that are hard to address?
3. What steps do you take to ensure patients are prepared for life after rehabilitation?
What facilitates or impedes this process?
How long does it take to complete the necessary steps?
4. How do you collaborate with other health‐care professionals?
Which health‐care professionals do you mostly collaborate with?
How does this collaboration, if any, help?
5. Do you feel that (Health‐care Centre) has effective strategies to allow patients to self‐manage after pulmonary rehabilitation?
What is working well?
What can be improved?
6. What advice can you give patients about self‐management after pulmonary rehabilitation?

Interviews ranged from 30 to 60 minutes. Following each interview, researchers recorded reflexive notes highlighting key concepts. Clinical data from the patient's medical records (eg, pulmonary function) and data from a socio‐demographic and clinical questionnaire were also collected.

### 2.4 Data analysis

The data were analysed using an iterative process that began during data collection.^25^ Interviews continued until data saturation was reached (ie, no new concepts were identified in subsequent interviews).^26^, ^27^ Data analysis was guided by the Qualitative Analysis Guide of Leuven model, following an iterative and constant comparison approach.^25^ The model is divided into two stages: preparation for coding and actual coding.^25^ In the first stage of analysis, the audio file of each interview was transcribed verbatim and was reviewed to ensure accuracy and de‐identification of the data. The first four transcripts were read by eight research team members to develop a preliminary coding framework. In the second stage of analysis, the preliminary framework was applied to subsequent transcripts and codes were adjusted as needed.^25^ The final framework was applied to four new transcripts by multiple coders. Interrater reliability was established by comparing application of the codebook during in‐person meetings. Discrepancies were discussed and resolved by the team before the remaining transcripts were coded. All transcripts were coded using the software package NVivo 10 with the finalized framework. Codes were compared between inpatients, outpatients, and health‐care professionals to identify main themes. This iterative approach to analysis facilitated an in‐depth exploration of participants' experiences.

This study received ethics approval from the University of Toronto (#35485) and the Health‐care Centre's Research Ethics Board (#17‐014). Informed consent was received from all participants.

## 3 RESULTS

Ten patients with COPD and eight health‐care professionals participated in this study. Demographic characteristics of participants are presented in Table 3. Patients from the inpatient and outpatient pulmonary rehabilitation programmes were equally represented. Six patients were attending the maintenance programme at the time of their interview. Health‐care professionals included physical therapists/assistants, nurses, and physicians (n = 8). Most health‐care professionals worked in both inpatient pulmonary rehabilitation and outpatient pulmonary rehabilitation (n = 5), with a median of 6 years of experience in pulmonary rehabilitation.

**Table 3:** Participant socio‐demographic and clinical characteristics

**Individuals with COPD (n = 10)**	
Median age in years (range)	67.5 (53‐80)
Sex (n)	
Male	8
Female	2
Marital status (n)	
Married/Common‐law	4
Other	6
Education level (n)	
Less than high school	2
High school diploma	6
More than high school	2
Used mobility aids (n)	2
Used supplemental oxygen (n)	1
Pulmonary rehabilitation programme attended (n)	
Outpatient	5
Inpatient	5
Attended maintenance	6
Median FEV1/FVC median (range)	0.54 (0.42‐0.74)
Median MRC breathlessness score (range)	3 (2‐4)
**Health‐care professionals (n = 8)**	
Median age in years (range)	38 (25‐70)
Sex (n)	
Male	1
Female	7
Median years experience median (range)	11 (1‐49)
Area of practice in pulmonary rehabilitation (n)	
Inpatient	1
Outpatient	2
Inpatient and outpatient	5
Median years experience in pulmonary rehabilitation (range)	6 (1‐35)

Abbreviations: COPD, chronic obstructive pulmonary disease; FEV1/FVC, forced expiratory volume in 1 second‐to‐forced vital capacity ratio.

Four main themes relating to pulmonary rehabilitation and patients' transition home were identified: (a) pulmonary rehabilitation as a safe environment; (b) pulmonary rehabilitation as a highly structured environment; (c) contrasting perceptions of the role of pulmonary rehabilitation; and (d) dependency on pulmonary rehabilitation programmes. Participants discussed three key factors that influenced this transition: (a) social support at home, (b) application of self‐management strategies prior to discharge, and (c) patients' physical and mental health.

### 3.1 Experiences and perceptions of pulmonary rehabilitation and the impact on transitions

#### 3.1.1 Pulmonary rehabilitation as a safe environment

Most patients and health‐care professionals had a positive view of the pulmonary rehabilitation programme. Patients described it as supportive and ‘extremely valuable’. Health‐care professionals also had a favourable view of the programme as it was thought to ‘produce good outcomes’. Both groups noted that rehabilitation provided a ‘comforting’ and ‘safe environment’ that was difficult to reproduce in a community setting. Patients highlighted that health‐care professionals were ‘very nice people’ who provided ‘a friendly face’ and were ‘open to any questions’. However, a positive view of pulmonary rehabilitation did not always lead to an easier or better experience transitioning home, as experiences depended on patients' ability to incorporate the skills from pulmonary rehabilitation into their everyday lives.

I would recommend [pulmonary rehabilitation] to anybody that needs it; but you only get out of it what you put into it. (P10, Patient)

#### 3.1.2 Pulmonary rehabilitation as a highly structured environment

Many participants in both groups described pulmonary rehabilitation, especially the inpatient programme, as being highly structured. Although some patients described their experience as feeling ‘institutionalized’ or ‘quasi‐militaristic’, they explained that the consistent scheduling allowed them to develop routines. In addition, ‘constant monitoring’ and ‘frequent reminders’ helped patients adhere to their exercises during the programme. Health‐care professionals described the inpatient programme as ‘all consuming’, ‘all encompassing’, and ‘comprehensive’; however, they also felt that it did not adequately prepare patients for the transition home. Most inpatients expressed a fear of transitioning to an unstructured home environment.

It was scary to go home because you don't have the structure… [In the inpatient program] you don't have to think about anything, [except] following what they are telling you to do. (P08, Patient)

In comparison, outpatients and health‐care professionals reported transitions were ‘simpler’ and ‘easier’ for outpatients because they could practice self‐management at home before being discharged.

[For inpatients], it's a little bit like boot camp. The patients come in, they exercise, go to education, that's all they really have to worry about. And then when they go home, all of a sudden, they have to do their grocery shopping, laundry, prepare meals and I think it's a big transition going from inpatient programming to home. (C01, Health‐care Professional)

#### 3.1.3 Contrasting perceptions of the role of pulmonary rehabilitation

Health‐care professionals and patients had different perceptions of the role of pulmonary rehabilitation. Health‐care professionals perceived pulmonary rehabilitation as a means of teaching patients exercises and self‐management strategies to help them to cope independently with their COPD. They acknowledged that more ‘problem‐solving’ and ‘independence’ should be incorporated into the programme to facilitate patients' transitions. Health‐care professionals also identified challenges with communicating the importance of behaviour changes. For example, they believed that patients viewed pulmonary rehabilitation as a ‘discrete intervention’ with a finite end date, rather than a way to learn strategies that can be applied outside of the programme. When asked about the role of pulmonary rehabilitation, patients inconsistently understood the goal of pulmonary rehabilitation. Even though a small number of patients understood the purpose (ie, facilitate the integration of home exercises and self‐management strategies into their daily lives), they often reported they were unable to maintain these lifestyle changes, particularly completing their daily home exercise programmes.

I know [breathing exercises] help me out, but hell, I don't do them. I don't know why. It's stupid. (P04, Patient)

In contrast, most patients viewed pulmonary rehabilitation as a ‘one‐stop‐shop’ that could ‘fix’ all their health concerns. For example, patients were reported to seek consultation on issues outside of their COPD such as acid reflux or joint issues. It was suggested by health‐care professionals that this occurred more frequently with patients attending the maintenance programme post‐discharge.

Sometimes … you end up being more of like a sounding board, they might have other health issues they are not quite sure about, they don't know where to go to. (C01, Health‐care Professional)

#### 3.1.4 Dependency on pulmonary rehabilitation programmes

Patients' perceptions of pulmonary rehabilitation as an intervention for all their health needs often led them to rely on the programmes. Many patients reported an increased confidence in their ability to manage acute illness following pulmonary rehabilitation; however, this confidence seemed to be partly rooted in the belief that the transition home was not permanent, and they would be readmitted to the programme as needed.

I had been six years in the maintenance program… then I got sick. My doctor [said], okay, you're well enough, we'll put you back in the rehab program. So, I imagine if I keep getting sick, I'm going to keep getting back into the rehab program. (P06, Patient)

Most health‐care professionals stated that many of the patients who attend the maintenance programme develop a dependency that hindered their ability to transition home. Although patients did not report having a dependency, there appeared to be a reliance on the maintenance programme for completing exercises and for social support.

Maintenance program has been a pool into which people dive without an anticipation of the time that they're going to leave… that wouldn't serve as a transition point, that's just a new place to live. (C02, Health‐care Professional)

### 3.2 Factors affecting transitions

#### 3.2.1 Social support

Many health‐care professionals and patients identified social support at home as a key factor for successful transitions from pulmonary rehabilitation to home. Health‐care professionals reported that those with less social support tended to express feeling ‘fearful’ and ‘nervous’ about going home. They emphasized the importance of patients having supportive individuals who understand COPD and its management. Patients also reported that social support at home encouraged accountability and motivation to complete daily exercises.

On my own, there's a lot of a chance that some days…I can't be bothered, whereas if somebody else is going to be with you, you don't want to let them down. (P03, Patient)

Have [family] involved in that meeting so that…they know what you're recommending so they can keep the patient accountable and be that support person. (C03, Health‐care Professional)

Moreover, social support from peers with COPD was identified as an important facilitator of transitioning from pulmonary rehabilitation to home through ‘camaraderie’, ‘understanding’, ‘moral support’, and a ‘push’ to exercise. These benefits were reported more frequently by patients attending the maintenance programme.

#### 3.2.2 Application of self‐management strategies prior to discharge

Most participants identified having the opportunity to apply the strategies they learned in pulmonary rehabilitation at home, before their discharge, as an important facilitator for successful transitions. All participants agreed that the outpatient programme provided more opportunities to apply learned skills at home and practice problem‐solving. This allowed patients to identify issues to be addressed in future sessions and facilitate their transition home.

It's a good chance for them to practice… they might have to learn some other techniques if the techniques they've learned already don't work out for them so well at home. (C06, Health‐care Professional)

The strategies most frequently reported as being useful in daily life included pacing, managing shortness of breath, and modifying daily activities. These components of patient education applied to functional tasks that were important to patients.

Once you stop and think about it and then apply it, it makes your life so much easier and it gives you back your life, because I didn't have one before. (P08, Patient)

Patients reported applying strategies less frequently when it was considered ‘impractical’ or ‘irrelevant’ to their everyday lives. Although patients realized the importance of breathing exercises and physical activity, they often reported infrequently completing these at home. Explanations often involved lack of motivation, inability to access resources such as a gym, or challenges with time management and prioritization of activities.

Unfortunately, I don't use [breathing exercises] … rather than being in the “have to do” file, it's in the “should do” file. (P03, Patient)

It's not doable. It just isn't. With all the other things that one has to do on a daily basis… it demands too much, and I think it's unrealistic. (P05, Patient)

#### 3.2.3 Patients' physical and mental health

Most patients reported a decline in their health status leading up to their admission to pulmonary rehabilitation; however, many patients expressed an improved ability to manage everyday activities after the completion of their inpatient or outpatient programme. This was often attributed to ‘increased lung function’, ‘better breathing’, and an ‘increased ability to conserve energy’.

Both patients and health‐care professionals reported that acute exacerbations hindered patients' ability to maintain the benefits of pulmonary rehabilitation after being discharged.

The other big barrier is if they get sick right after they leave. They get a chest infection and exacerbation, they can't exercise, they don't feel well and then it's hard for them to get started again. (C01, Health‐care Professional)

Patients also reported musculoskeletal comorbidities unrelated to COPD to be another significant barrier to completing activities and exercises at home.

I've got exercises to fix that shoulder I hope eventually. But [my shoulder pain inhibits] doing the breathing exercises which I should be doing every day. (P05, Patient)

Health‐care professionals acknowledged that musculoskeletal comorbidities complicated patients' transitions to home but expressed an inability to manage these conditions in pulmonary rehabilitation due to limited time and resources.

Many patients and health‐care professionals agreed that COPD is often associated with mental health challenges. Anxiety about shortness of breath and fear of disease progression hindered the transition home.

Their concerns are often around the respiratory when they go home, but I find that in practice, their concerns are about all their medical concerns. It's not simply their breathing, their cough, their sputum, it's also their diabetes, their foot pain, their back pain, their digestion and other things that they were bringing up regularly that now they are concerned they won't have somebody to listen… (C02, Health‐care Professional)

Health‐care professionals and patients both highlighted that a positive outlook towards the programme and their condition facilitates the transition, while a sense of hopelessness acts as a barrier.

Once you started to the program and stopped feeling sorry for yourself and you gradually learn from the education that they provide and it just makes you stronger. It delays all your fears (P06, Patient)

## 4 DISCUSSION

In this study, we explored the experiences of transitions from pulmonary rehabilitation to home from the perspectives of persons with COPD and health‐care professionals. Although persons with COPD and health‐care professionals approached the issue of transitions in care from different perspectives, both groups agreed on four key themes related to the perceptions of, and experiences with, pulmonary rehabilitation as well as three factors that they felt affected transitions. Social support was highlighted as important for transitions because it increases motivation and accountability for patients to complete their home exercise programmes. Another important element was the opportunity to apply strategies learned in pulmonary rehabilitation in the home environment prior to discharge. Lastly, physical and mental health status played a key role in patients' capacity to self‐manage at home.

An effective pulmonary rehabilitation programme focuses on behaviour change to maintain its benefits, but this is challenging to implement in practice.^20^, ^21^ Most health‐care professionals spoke of the need for a greater focus on problem‐solving to better prepare patients for the unpredictable nature of everyday life at home. This idea is supported by Bourbeau and van der Palen who suggested that opportunities to practice and problem‐solve are required to develop self‐management skills, and it is inadequate to rely solely on patient education.^20^ Patients often had the perception that they could return to pulmonary rehabilitation at any time if needed, which suggests patients viewed the programme as a ‘safety net’ rather than a way to learn effective self‐management strategies.

The type of pulmonary rehabilitation—inpatient or outpatient—also influenced the opportunity to practice self‐management skills. The structured inpatient environment failed to take into account the unpredictable nature of life at home and seemed to create a susceptibility to programme dependency. Conversely, the outpatient programme has less imposed structure since patients do not stay at the centre, and these patients described a smoother transition in comparison with those in the inpatient programme. Further, for some outpatients, the transition period seemed to have been blurred because of the similarities with the maintenance programme. While the maintenance programme is intended to supplement patients' home exercise routines post‐discharge, some patients used the once a week session as their only weekly exercise. As a result, patients likely experienced a decline in function as this amount of exercise is insufficient for maintaining the benefits of pulmonary rehabilitation^23^ and does not meet the American College of Sports Medicine guidelines for COPD.^28^ Furthermore, a systematic review of the effects of supervised exercise programmes similar to the maintenance programme suggested that programmes that offered sessions 3 days per week or less were unable to maintain the benefits of pulmonary rehabilitation at 12 months post‐discharge.^23^ Patients relying on these weekly sessions suggest that pulmonary rehabilitation failed to induce lasting behaviour change and that patients may develop a dependency on pulmonary rehabilitation and its affiliated programmes. This may result in further functional decline when patients are eventually discharged from the maintenance programme.

Social support, such as having a caregiver at home, was identified in our study as an important facilitator of transitions. The benefits of social support to facilitate transitions (eg, reduce anxiety and fear) and self‐management are supported by previous studies.^13^, ^19^, ^29^, ^30^ DiNicola and colleagues examined social support and the impact on anxiety for patients with COPD. They found that patients surrounded by individuals with unsympathetic or insensitive behaviour had higher anxiety levels.^30^ Patients in our study indicated that social support increased their motivation to complete their daily exercise and that caregivers provided reminders to pace themselves during their daily activities. Chen and colleagues reported that persons living with a caregiver at home were more likely to participate in outpatient pulmonary rehabilitation programmes and had higher physical activity levels.^29^ Similarly, our study found that although adherence to home exercise programmes was relatively poor for our participants, those who reported exercising more frequently identified their family or caregivers as key motivators.

The opportunity to apply the skills from pulmonary rehabilitation was identified as a significant factor impacting transitions. Despite the fact that patients reported they understood the importance and benefits of exercise, they had difficulty continuing to exercise at home unless the exercises were incorporated into their daily routines. This was particularly true for inpatients as they were not living at home during pulmonary rehabilitation. Our findings are consistent with the literature, as daily exercise is infrequently achieved during the transition from pulmonary rehabilitation to home.^14^, ^16^, ^21^ Our results suggest that exercise adherence may be improved by making the exercises more relevant to the patients, thereby making it more intrinsically motivating to implement in daily life. Intrinsic motivation has previously been noted as an important aspect of maintenance of behaviour change in this population.^22^ Examples of relevant exercises in this study included activity modification, pacing, and managing shortness of breath.

Our study also identified that the physical and mental health status of patients (eg, arthritis, anxiety and depression) affected their overall care transitions and ability to self‐management. Previous studies have also shown psychological issues (eg, frustration, guilt, anxiety, and depression), as well as other comorbid conditions, to be barriers for self‐management.^19^, ^31^, ^32^ Specifically, patient anxiety and breathlessness were identified in a systematic review as common factors affecting patients' ability to self‐manage.^21^ Similarly, patients and health‐care professionals in this study identified anxiety related to shortness of breath as a common concern affecting patients' transitions.

Due to the progressive nature of COPD, exacerbations often impact an individual's ability to exercise and complete activities of daily living.^31^, ^32^, ^33^ Our study supports this finding, as exacerbations resulted in a more difficult transition because patients were unsure how to resume exercising after further functional decline. A unique finding from this study was that patients believed that they would be readmitted to pulmonary rehabilitation if their functioning decreased following an exacerbation. This suggests that, although pulmonary rehabilitation is designed to provide individuals with the tools to self‐manage during and after exacerbations, patients relied on the programme to solve more significant episodes of functional decline.

## 5 LIMITATIONS

A limitation of this study is the selection of participants who were English‐speaking‐only. It is possible that individuals who speak other languages may have different experiences with transitions. Additionally, of the ten persons with COPD, only one individual used supplemental oxygen; therefore, future research should aim to understand how oxygen needs influence transitions from rehabilitation to home.

## 6 CONCLUSION

The transition from pulmonary rehabilitation to home for patients with COPD can be a challenging experience. The perception of pulmonary rehabilitation, social support, changing health status, and the application of skills learned at pulmonary rehabilitation were identified as key factors that contribute to this experience. Understanding how these factors influence the transition will inform an individualized approach, preparing patients for life at home. This may include prescribing exercises that are more salient to patients, completing home visits to identify barriers prior to discharge, increasing autonomy in pulmonary rehabilitation, or implementing social support programmes in the community once patients have transitioned home. Future research is warranted to develop and evaluate interventions and programmes to improve the transition from a structured intensive rehabilitation to the community, which will likely improve the self‐management skills and quality of life for persons with COPD.

## CONFLICT OF INTEREST

The authors report no conflicts of interest. 

## Data Availability

Due to confidentiality and the nature of the consent obtained, the interview transcripts cannot be shared. For further information related to this data set, contact the senior author.
